# Intermittent hypoxia causes NOX2-dependent remodeling of atrial connexins

**DOI:** 10.1186/s12860-016-0117-5

**Published:** 2017-01-17

**Authors:** Joanna Gemel, Zihan Su, Alex Gileles-Hillel, Abdelnaby Khalyfa, David Gozal, Eric C. Beyer

**Affiliations:** 1 Department of Pediatrics, University of Chicago, 900 E. 57th St. KCBD 5152, Chicago, IL 60637 USA; 2 Present address: Williams College, Williamstown, MA USA; 3 Present address: Department of Pediatrics, Hadassah-Hebrew University Medical Center, Mt. Scopus, Jerusalem Israel

**Keywords:** Intermittent hypoxia, Obstructive sleep apnea, Atrial fibrillation, Gap junctions, Connexin

## Abstract

**Background:**

Obstructive sleep apnea has been linked to the development of heart disease and arrhythmias, including atrial fibrillation. Since altered conduction through gap junction channels can contribute to the pathogenesis of such arrhythmias, we examined the abundance and distributions of the major cardiac gap junction proteins, connexin40 (Cx40) and connexin43 (Cx43) in mice treated with sleep fragmentation or intermittent hypoxia (IH) as animal models of the components of obstructive sleep apnea.

**Results:**

Wild type C57BL/6 mice or mice lacking NADPH 2 (NOX2) oxidase activity (gp91phox(−/Y)) were exposed to room air or to SF or IH for 6 weeks. Then, the mice were sacrificed, and atria and ventricles were immediately dissected. The abundances of Cx40 or Cx43 in atria and ventricles were unaffected by SF. In contrast, immunoblots showed that the abundance of atrial Cx40 and Cx43 and ventricular Cx43 were reduced in mice exposed to IH. qRT-PCR demonstrated significant reductions of atrial Cx40 and Cx43 mRNAs. Immunofluorescence microscopy revealed that the abundance and size of gap junctions containing Cx40 or Cx43 were reduced in atria by IH treatment of mice. However, no changes of connexin abundance or gap junction size/abundance were observed in IH-treated NOX2-null mice.

**Conclusions:**

These results demonstrate that intermittent hypoxia (but not sleep fragmentation) causes reductions and remodeling of atrial Cx40 and Cx43. These alterations may contribute to the substrate for atrial fibrillation that develops in response to obstructive sleep apnea. Moreover, these connexin changes are likely generated in response to reactive oxygen species generated by NOX2.

**Electronic supplementary material:**

The online version of this article (doi:10.1186/s12860-016-0117-5) contains supplementary material, which is available to authorized users.

## Background

Sleep disordered breathing and, more specifically, obstructive sleep apnea (OSA) have been linked to a variety of cardiovascular diseases including hypertension, coronary artery disease, arrhythmias, heart failure, and stroke [1–3]. Atrial fibrillation is the most common cardiac arrhythmia, and its prevalence has been linked to OSA in many epidemiologic and clinical studies [4]. The prevalence of OSA is increased in AF patients, and conversely the prevalence of AF is increased in patients with OSA [5–7]; moreover, the risk of AF increases with the presence and severity of intermittent hypoxia during sleep [8–11]. Multiple mechanisms have been suggested for how sleep disordered breathing predisposes to AF, including sympathetic hyperactivity, oxidative stress, inflammation, and left atrial stretch with myocardial remodeling (reviewed in [12]).

The pathogenesis of arrhythmias often involves structural changes such as alterations of intercellular junctions and fibrosis. AF is initiated by triggers (e.g., rapidly firing ectopic foci located inside the pulmonary veins) and is sustained by the underlying presence of an abnormal atrial tissue substrate [13, 14] that is largely determined by the abundance and distribution of intercellular channels contained within atrial gap junctions [15, 16]. Gap junction channels allow the exchange of ions (and small molecules) between adjacent cells, and are critical for normal electrical conduction in all regions of the heart, and remodeling of gap junction organization and expression of the subunit proteins (connexins) is a common feature of human heart diseases associated with arrhythmias [17]. Atrial myocardium contains two major connexins of similar abundance, namely Cx40 and Cx43, while ventricular myocytes predominately express Cx43 [18].

Animal models that can be exploited to understand the relationships between OSA and cardiac arrhythmias are now being developed and analyzed. Here, we utilized two mouse model to mimic the components of OSA: sleep fragmentation (SF) and intermittent hypoxia (IH) in which chronic intermittent hypoxia (IH) closely mimics oxyhemoglobin desaturation patterns associated with OSA [19]. These mice show many of the abnormalities observed in patients with OSA and have been extremely useful for examining the pathophysiology of several end-organ morbidities [20–24]. Many of the abnormalities in these animals appear to derive from reactive oxygen species generated via excessive activation of NADPH Oxidase 2 (NOX2) [25–27].

Therefore, to advance our understanding of the cardiac remodeling in response to OSA, we studied the hearts of mice exposed to chronic SF or IH, and compared them to control mice exposed to normoxic environments. We examined the levels and distributions of the connexins that form atrial and ventricular gap junctions using a strategy similar to that which we have previously used to study the connexin alterations in atrial tissue from people with isolated atrial fibrillation [28].

## Results

We initially compared the histologic appearance of atrial and ventricular tissue sections from IH or RA-exposed C57BL/6 J mice. We found no obvious differences in cell size or shape. To assess potential fibrosis, we quantified the abundance of extracellular space by staining with fluorescent WGA, which has similar staining properties to Masson’s trichrome, used to detect collagen [29]. Images of atrial or ventricular sections from mice treated with RA or IH showed similar patterns and extents of staining with WGA-Texas Red-X (example shown in Additional file 1). We found no significant differences in the percentages of tissue area that reacted with WGA between control and IH in atria (12% for RA vs. 11% for IH) or ventricle (9.7% for RA vs. 9.5% for IH) *(p > 0.05, Student’s t test*, *n* = 10 for RA, *n* = 11 for IH). Therefore, we concluded that the extent of fibrosis was not affected by exposure to IH.

**Fig. 1:**
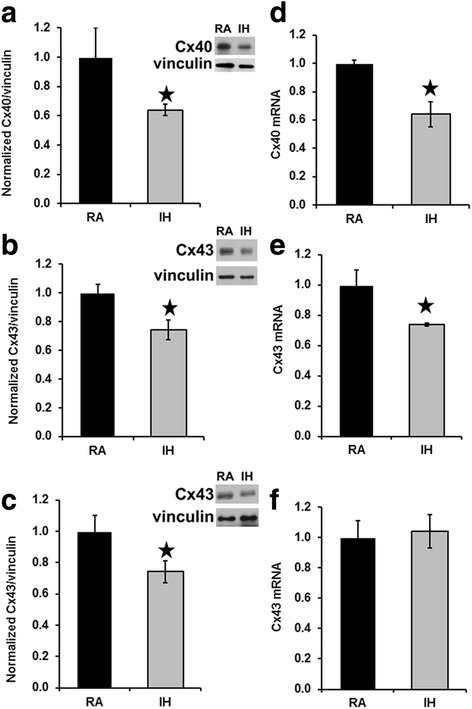
Connexin levels are reduced in homogenates of atrium (**a**, **b**) and of ventricle (**c**) in samples prepared from wild type C57BL/6 J mice exposed to intermittent hypoxia. Homogenates were prepared from atria (**a**, **b**) or ventricles (**c**) of mice exposed to room air (RA) or to intermittent hypoxia (IH). Cx40 (**a**) and Cx43 (**b**, **c**) were detected by immunoblotting. Gels were loaded with equal amounts of total protein (10 μg for A, 1 μg for B, C). Blots were also reacted with antibodies directed against vinculin (as a loading control). Graphs show the amounts (mean ± SEM) of immunoreactive connexin in control (RA) and IH mice determined by densitometry (adjusted for vinculin and normalized to the mean control values). Representative blots are shown on the right. The abundances of Cx40 and Cx43 were significantly less in all IH groups (grey bars) as compared with corresponding RA controls (black bars; *, p < 0.05, Student’s t test, n = 6 for atrium; *n* = 9 for ventricle)

In order to determine any alterations in the levels of connexins in these mice, we prepared homogenates from atria and from ventricles of mice treated with RA or IH, and detected Cx40 or Cx43 by immunoblotting (Fig. 1). We found that the levels of both connexins were significantly reduced in atria (Cx40 and Cx43) and ventricles (Cx43) of IH-exposed mice (Fig. 1). Atrial Cx40 levels in IH mice were reduced to 64% of control values, while levels of Cx43 in either atrium or ventricle of mice treated with IH were reduced to 72% of values from mice exposed to RA.

**Fig. 2:**
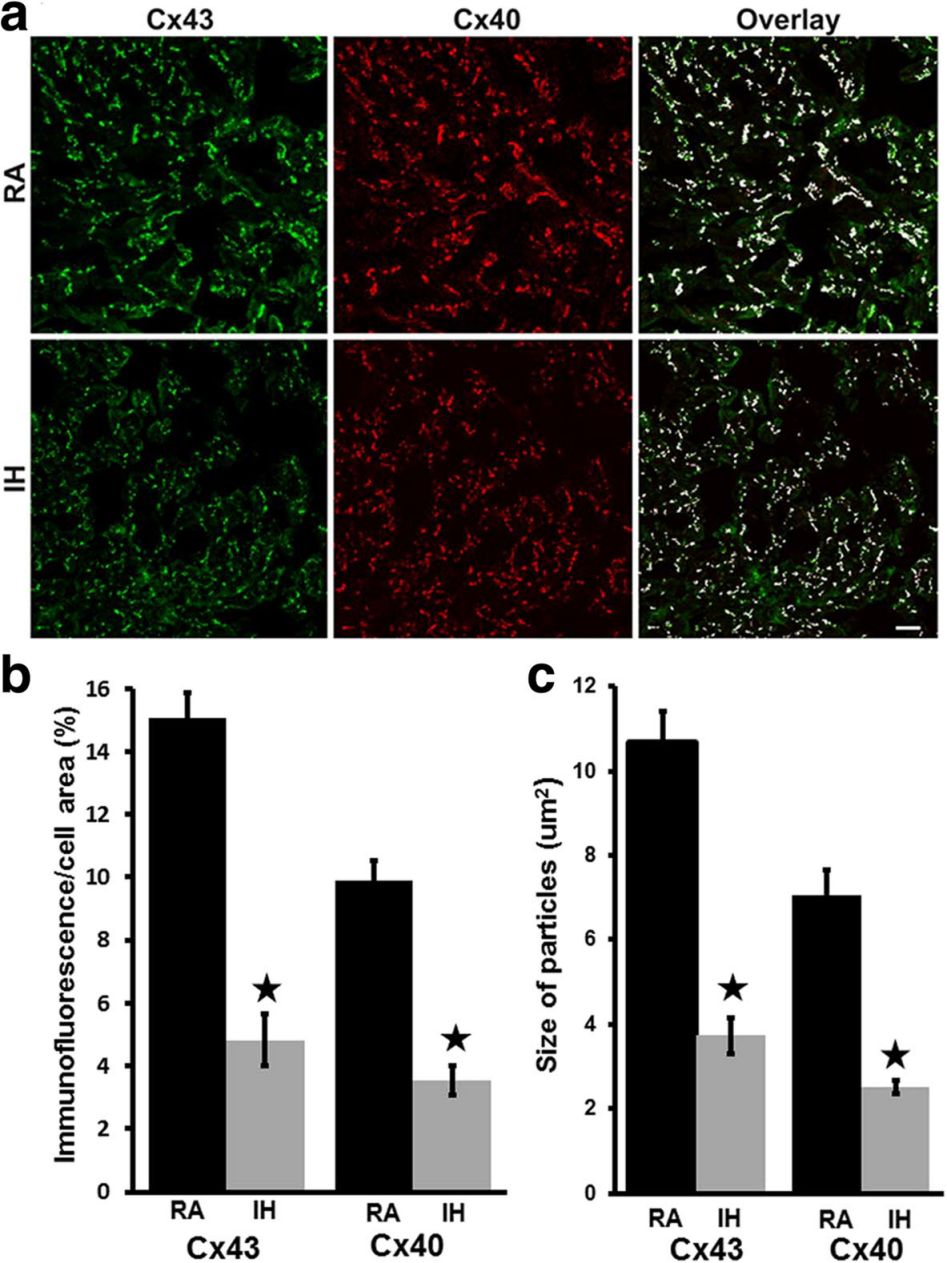
The abundance and sizes of Cx40- and Cx43-containing gap junctions are reduced in atria of wild type C57BL/6 J mice exposed to intermittent hypoxia. Cx43 and Cx40 were localized by double label immunofluorescence in frozen sections of atria from mice treated with RA or IH. **a** Representative photomicrographs show localization of Cx43 (green), Cx40 (red) and their co-localization (white). Bar, 20 μm. Both Cx43 and Cx40 are detected in RA and IH samples; levels of each appear decreased in the IH images; but, the extent of their overlap did not differ between RA and IH. (See text for quantitation of overlap.) **b** Graph depicts the quantitation of the abundance of Cx43- and Cx40-containing gap junctions in these samples (based on image analysis as described in Material and Methods). The abundances of both Cx43 and Cx40 immunofluorescence per cell area were significantly reduced in atria from IH mice (*, p < 0.05, *Student’s t test, n* = 18 for RA, black bars; *n* = 16 for IH, grey bars). **c** Graph depicts the quantitation of the sizes of Cx43- and Cx40-containing gap junctions in these samples. The sizes of Cx43 or Cx40 immunoreactive particles were significantly reduced in atria from IH mice (*, p < 0.05, Student’s t test, n = 18 for RA, black bars; *n* = 16 for IH, grey bars)

We studied the abundance and distribution of Cx43 and Cx40 in the atria of mice treated with RA or IH. Multiple frozen sections were cut from each sample, and Cx43 and Cx40 were simultaneously detected by double label immunofluorescence. As illustrated by the examples shown in Fig. 2a (top panels), immunoreactivity corresponding to each of the connexins was abundantly detected in discrete spots (corresponding to gap junction plaques) in control atria. As shown in the overlay image, the two connexins co-localized extensively with a few spots containing only Cx43 and very rare spots containing only Cx40. In atrial sections from mice treated with IH, Cx43 and Cx40 also were extensively co-localized. We quantified this overlap in multiple samples as we have done previously [28], and found no differences in the Mander’s coefficients that quantify the overlap of Cx40 on Cx43 (0.66 for RA vs. 0.68 for IH) or of Cx43 on Cx40 (0.86 for RA vs. 0.76 for IH). However, the abundance of staining for either Cx43 or Cx40 appeared dramatically reduced in the IH samples. We quantified these changes by assessing the percentage of cellular area corresponding to gap junctions (which was reduced from 15.0 to 4.8% for Cx43 and from 9.9 to 3.5% for Cx40). Moreover, the size of immunoreactive particles (a surrogate measure of gap junction size) was reduced (from 10.7 to 3.7 μm^2^ for Cx43 and from 7.1 to 2.5 μm^2^ for Cx40).

**Fig. 3:**
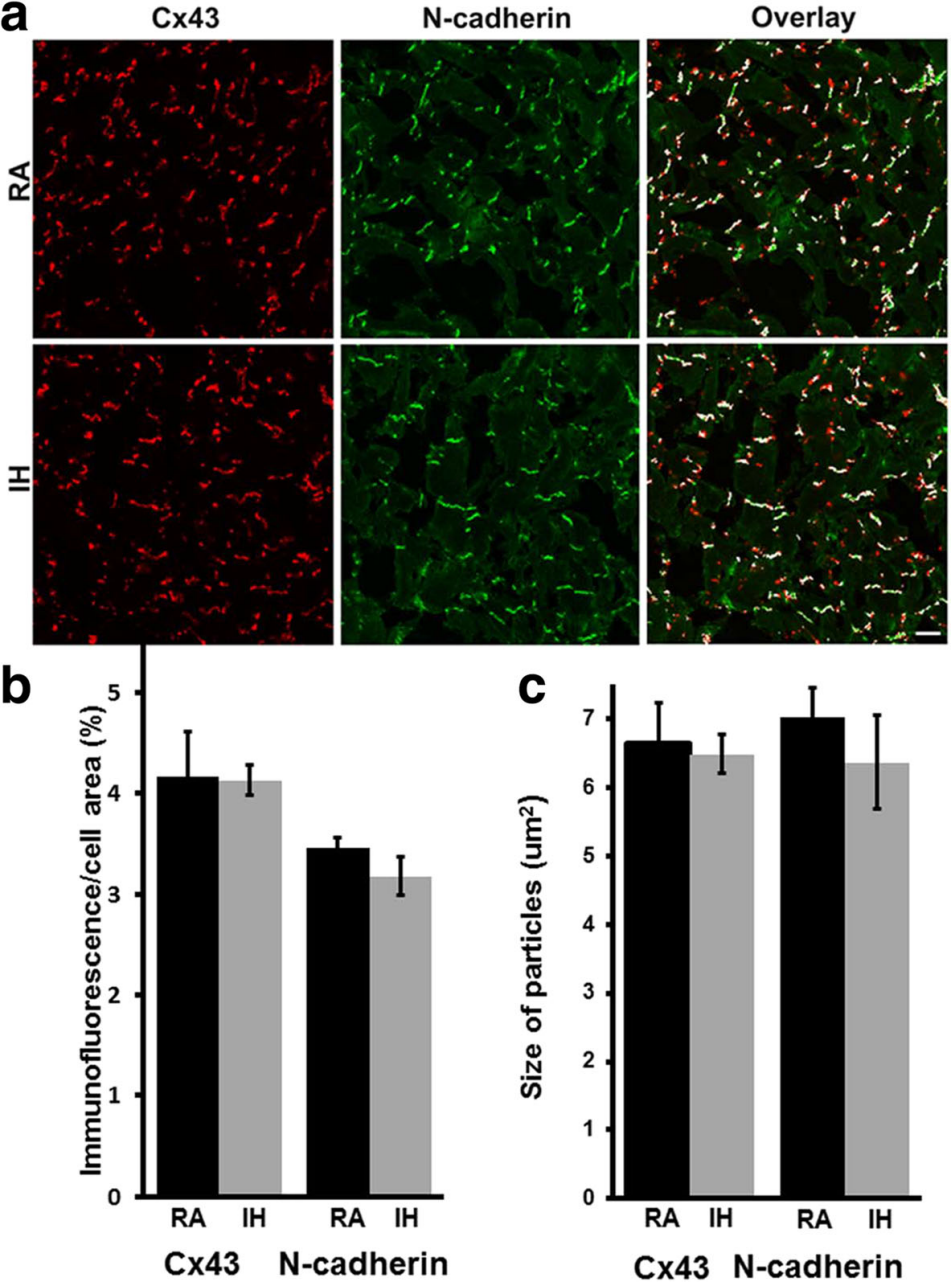
The abundance and distribution of Cx43 and of N-cadherin immunoreactive staining are not altered in ventricles of mice exposed to intermittent hypoxia. Cx43 and N-cadherin were localized by double label immunofluorescence in frozen sections of ventricles from wild type C57BL/6 J mice treated with RA or IH. **a** Representative photomicrographs show localization of Cx43 (red) or N-cadherin (green) and their co-localization (white). Bar, 20 μm. Cx43 and N-cadherin are detected in both RA and IH samples; levels of each, their distributions, and their overlap do not appear very different between RA and IH. **b** Graph depicts the quantitation of the abundance of Cx43- and N-cadherin-immunoreactivity in these ventricular samples. The abundances of Cx43 and N-cadherin did not differ significantly between RA (black bars) and IH treated mice (grey bars; *p >0.05, Student’s t test)*. **c** Graph depicts the quantitation of the sizes of ventricular Cx43- and N-cadherin-containing immunoreactive objects in these samples. The sizes of Cx43 or N-cadherin immunoreactive particles did not differ significantly between RA (black bars) and IH treated mice (grey bars; *p >0.05, Student’s t test). n* = 5 for RA, *n* = 3 for IH

We conducted a similar immunohistological examination of the abundance and distributions of Cx43 in ventricle (Fig. 3). We performed double label immunostaining with antibodies to N-cadherin to allow a comparison with the abundance and distributions of an adhesive component of the intercalated discs. We did not observe changes in the distributions of either Cx43 or N-cadherin between ventricular sections of RA or IH mice nor in the overlap of the immunostaining for these two molecules. The extent of Cx43 and of N-cadherin immunostaining (as a percentage of cell area) did not differ between RA and IH, nor did the sizes of particles stained with the two antibodies (Fig. 3b). These findings suggested that in the ventricles (unlike the atria), IH did not lead to significant remodeling of gap junctions.

**Fig. 4:**
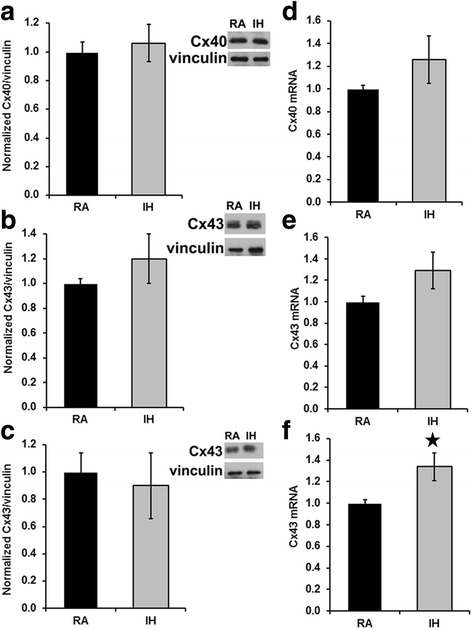
In NOX2-null mice, connexin levels in atrium and in ventricle are not affected by exposure to intermittent hypoxia. Homogenates were prepared from atria (**a**, **b**) or ventricles (**c**) of NOX2-null mice exposed to room air (RA) or exposed to intermittent hypoxia (IH). Cx40 (**a**) and Cx43 (**b**, **c**) were detected by immunoblotting. Gels were loaded with equal amounts of total protein (10 μg for A, 1 μg for B,C) and were also blotted with antibodies directed against vinculin (as a loading control). Graphs show the amounts (mean ± SEM) of immunoreactive connexin in control (RA) and IH mice determined by densitometry (adjusted for vinculin and normalized to the mean control values). Representative blots are shown on the right. The abundances of Cx40 and Cx43 did not differ significantly between RA (black bars) and IH groups (grey bars; *p > 0.05, Student’s t test). n* = 3 for atrium; *n* = 3 for ventricle)

**Fig. 5:**
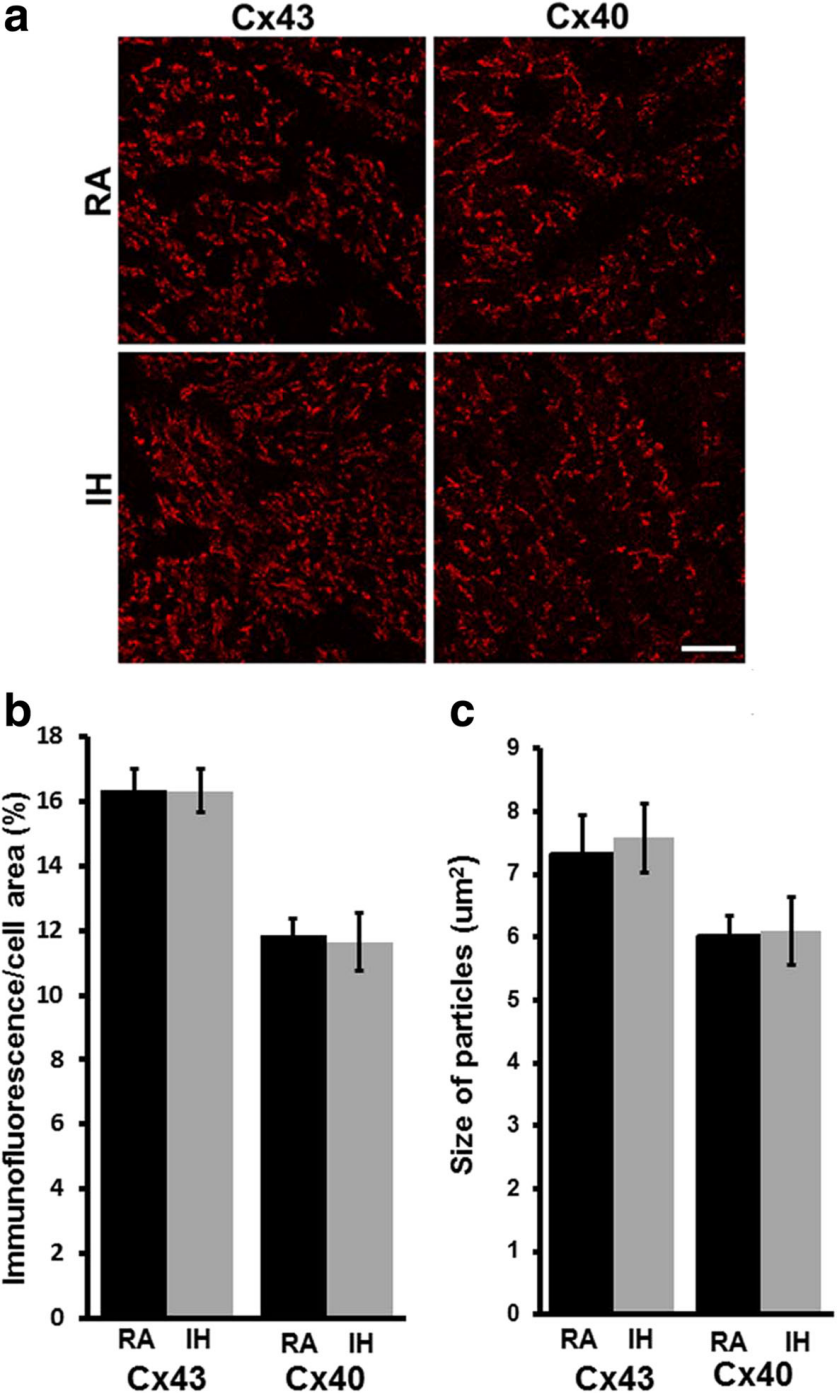
In NOX2-null mice, the abundance and sizes of Cx40- and Cx43-containing gap junctions are not altered in atria of mice exposed to intermittent hypoxia. Cx43 and Cx40 were localized by immunofluorescence in frozen sections of atria of NOX2-null mice treated with RA or IH. (A) Representative photomicrographs are shown for each of these tissues and conditions. Bar, 20 μm. (B) Graph depicts the quantitation of the abundance of Cx43- and Cx40-immunoreactivity in atrial samples. The abundances of Cx43 and Cx40 did not differ significantly between RA (black bars) and IH treated mice (grey bars; *p >0.05, Student’s t test)*. (C) Graph depicts the quantitation of the sizes of atrial Cx43- and Cx40-containing immunoreactive objects in these samples. The sizes of Cx43 or Cx40 immunoreactive particles did not differ significantly between RA (black bars) and IH treated mice (grey bars *p >0.05, Student’s t test). n* = 14 for all treatments

Many of the pathological changes induced by IH can be attributed to ROS generated by NOX2. Therefore, we also examined the connexin abundance and distributions in NOX2-null mice treated with RA or IH. Unlike our findings in the wild type C57BL/6 J mice, we found no significant differences in Cx40 or Cx43 levels in atrial or ventricular homogenates prepared from atria or ventricles of NOX2-null mice treated with RA or IH (Fig. 4). Moreover, in immunostained sections of atria from NOX2-null mice, there were no differences in the distributions of Cx43 or Cx40 between animals treated with RA or IH (Fig. 5a). Quantitation of the percentage of cellular areas occupied by gap junctions and of gap junction size showed no differences between RA and IH mice (Fig. 5 b, c). Thus, in the absence of NOX2 expression, IH did not elicit a reduction or remodeling of cardiac connexins.

## Discussion

In this study, we have shown that mice exposed to IH exhibit significant alterations of cardiac connexins and gap junctions. It is likely that these alterations contribute to the pathogenesis of arrhythmias induced by chronic IH. The most consistent and significant changes that we observed were in the atria of these animals where the abundance of both Cx40 and Cx43 was reduced, as was the total area and individual sizes of gap junctions containing these connexins.

The reductions of Cx40 and Cx43 might likely predispose to atrial arrhythmias. Iwasaki et al. [30] recently showed in a similar rodent model that IH increased the inducibility and duration of AF. Our observed reductions of atrial gap junction channels would likely contribute to the substrate for this arrhythmia. It is also likely that IH might also induce similar reductions of atrial gap junctions and predispose to AF in humans as well. Many investigators have found reductions or remodeling of Cx40 and/or Cx43 associated with AF (reviewed by [31]). We have observed major reductions in Cx40 in tissues from patients with lone AF [28]. As noted above, many studies have linked OSA and AF [5–11]. Indeed, prior investigators have suggested that OSA may explain the etiology of AF in many patients previously labeled as “lone” AF [32].

In our study, reductions of cardiac Cx40 or Cx43 were not observed in NOX2-null animals. Therefore, it is likely that reactive oxygen species (ROS) generated by NOX2 are responsible for the connexin remodeling. This is an important link between IH/OSA and the pathogenesis of AF. Previous studies of these mice have shown that excessive activation of NOX2 in response to IH contributes to pathologies of the brain, adipose tissue, and inflammatory system [25–27]. In both animal models and humans, increased atrial oxidative stress has been implicated in initiating and sustaining AF. NOX2 is the main source of ROS in human atrial myocytes [33]. Moreover, AF develops spontaneously in mice with cardiac-specific overexpression of a constitutively active Rac1, which activates NOX2 [34]. Therefore, targeting this NADPH Oxidase and the related pathways as proposed to address both OSA [25, 26] and AF [35] might block the atrial connexin remodeling.

We also observed that exposure to IH produced substantial reductions of Cx43 in ventricle as determined by immunoblotting. However, we did not detect any alterations of Cx43 abundance or distribution in ventricle by immunofluorescence. Because ventricular gap junctions are very large, often containing thousands of connexin channels, a 28% reduction of Cx43 might be undetectable by immunofluorescence. Regardless, we saw no evidence of remodeling or alterations of the localization of Cx43 as compared to another component of the ventricular intercalated discs (N-cadherin), this argues that the Cx43 reductions might not affect the anisotropy of ventricular conduction. The IH-induced reduction of ventricular Cx43 was not observed in NOX2-null mice. This suggests that ventricular Cx43 is another one of the many components of this tissue that are pathologically affected by NOX2 generated ROS [36].

## Conclusions

Exposures to intermittent hypoxia (but not sleep fragmentation) mimicking obstructive sleep apnea are accompanied by significant alterations in atrial connexins mediated, at least in part, by induction of oxidative stress pathways. These connexin changes may underlie the increased propensity for patients with OSA to develop atrial arrhythmias.

## Methods

### Mice and heart tissues

Eight-week-old male C57BL/6 J mice or hemizygous gp91phox-/Y (B6.129SCybb tm1Din/J) mice (NOX2-null) (Jackson Laboratories, Bar Harbor, ME) were exposed to intermittent hypoxia (IH) with alternating 90-s cycles (21% FiO_2_ followed by 6% FiO_2_, 20 cycles/h) during daylight for 12 h/for 6 weeks. A control group was maintained in continuous circulating room air (21% FiO_2_; RA). Mice were randomly assigned to either IH or RA exposures [19].

After the completion of IH or RA exposures, the mice were sacrificed (by introduction of 100% carbon dioxide into a euthanasia chamber) and hearts were immediately dissected. Atria were separated from the ventricles; tissues were flash frozen in liquid nitrogen and stored in −80 °C until assayed.

The experimental protocols were approved by the Institutional Animal Use and Care Committee and were in close agreement with the National Institutes of Health Guide in the Care and Use of Animals. All efforts were made to minimize animal suffering and to reduce the number of animals.

### Antibodies and fluorescent lectin

Cx40 was detected using rabbit polyclonal antibodies directed against the carboxy-terminal domain of Cx40 (cat. no 36–4900 Life Technologies, Grand Island, NY). It was used at 1:200 dilution for immunofluorescence and at 1:1000 dilution for immunoblotting. Cx43 was detected using a mouse monoclonal antibody directed against amino acids 252–270 (MAB 3067, Millipore/Chemicon, Billerica, MA) for immunofluorescence at dilution 1:200 or using rabbit polyclonal antibodies directed against amino acids 363–382 of human/rat Cx43 (C6219, SIGMA Chemical Company, St. Louis, MO) at 1:1000 dilution for immunofluorescence and at 1:12,500 dilution for immunoblotting. Mouse monoclonal anti-N-cadherin antibodies (cat. no 33–3900 Life Technologies, Grand Island, NY) were used at 1:200 dilution for immunofluorescence. Rabbit monoclonal anti-vinculin antibodies were obtained from abcam (cat. no ab129002 Cambridge, MA) and used at 1: 10,000 for immunoblotting were 1 μg of protein was loaded or at 1:3000 dilution were 1 μg of protein was loaded to verify accuracy of protein loading after treating blots with Restore Plus Western blot stripping buffer (Thermo Fisher Scientific Inc., Waltham, MA). Cy3-conjugated goat anti-rabbit or Alexa Fluor488- conjugated goat anti-mouse IgG and HRP-conjugated goat anti-rabbit or anti-mouse IgG antibodies were obtained from Jackson ImmunoResearch (West Grove, PA). Wheat Germ Agglutinin (WGA)-Texas Red®-X conjugate (Life Technologies) was used at 1:200 dilution.

### Immunoblot analysis

Heart tissue was disrupted in a glass Kontes homogenizer using 25–100 μl of 50 mM Tris–HCl (pH 8.0) buffer containing 150 mM NaCl, 1% Triton ×-100, 0.02% sodium azide, 50 mM sodium fluoride, 0.5 mM sodium orthovanadate, and Roche mini EDTA-free protease inhibitors (Roche Applied Science, Indianapolis, IN) (one tablet per 5 ml of lysis buffer) [18].

The protein concentrations of homogenates were determined using the method of Bradford (1976) [37] (Bio-Rad, Richmond, CA). Aliquots containing 1 μg of protein for the detection of Cx43 or 10 μg for the detection of Cx40 were separated by SDS-PAGE on 10% polyacrylamide gels and blotted onto Immobilon-P membranes (Millipore, Bedford, MA) [18].

ProSieve QuadColor Protein Markers standards (Lonza Rockland, Inc; ME) were used to calibrate the gels. Immunoblots were developed with ECL chemiluminiscence reagents (GE Healthcare Biosciences) and exposure to X-ray film.

### Immunohistochemistry and fluorescent-lectin staining

Nine to twelve cryosections (8 μm thick) of atrium or ventricle sample were put on one slide. Slides were fixed with 3% paraformaldehyde for 15 min and then blocked twice for 15 min and once for 30 min at room temperature using buffer composed of 10% heat inactivated normal goat serum in PBS containing 0.75% Triton X-100. Slides were subsequently incubated with primary antibodies diluted in blocking solution overnight at 4 °C. Following incubation with primary antibodies, slides were washed six times for five-minutes with PBS. Each slide was then incubated with secondary reagents at room temperature for 75 min. For WGA staining, the blocking step was omitted; fixed sections were incubated for 10 min with WGA-Texas Red-X. Afterwards, the slides were extensively rinsed again with PBS. Each slide was mounted with a cover slip using Prolong Gold anti-fade reagent (Life Technologies). Slides were sealed and stored in darkness at 4 °C.

### Confocal microscopy

A Leica TCS SP2 laser scanning confocal microscope was used to examine the distributions and co-localization of Cx40 and Cx43 in mouse atrium or a connexin and N-cadherin in mouse atrium and ventricle sections. For each sample, at least five images were captured using the 63× immersion objective. For co-localization studies each image included 3 channels: Cy3, Cy2, and differential interference contrast (DIC). Snapshots of images from each channel as well as all combinations of channel overlap were recorded. For all samples, the laser voltage at which images were captured in each filter was kept constant in order to minimize differences in connexin detection between samples. Images were acquired using sequential laser scanning to avoid bleed-through. The plane and quality of sections were evaluated using DIC, and flat, even sections were subsequently photographed. All images were captured within one week of sectioning and staining. Images of sections stained with WGA-Texas Red-X were captured using a 20× objective (at least 5 images per sample).

### Colocalization analysis

Image analysis for colocalization of Cx43 and Cx40 (or connexins and N-cadherin) was performed using the JACoP plugin for Image J software (http://rsb.info.nih.gov/ij/) as described by Bolte and Cordelières, 2006. [38]. The program analyzes image pairs of the same visual field using a compilation of general colocalization indicators. In the present study, each image pair consisted of one image from the Cy3 channel and one image from the Cy2 channel. All images were acquired in such a way as to minimize variation in connexin staining intensity within each channel. Image pairs were analyzed using Mander’s coefficients, which do not use average pixel intensity in evaluating colocalization. Mander’s coefficients are, however, very sensitive to non-specific staining or “background.” In order to exclude such non-junctional staining from the Mander’s calculation, a threshold level of pixel intensity was measured and subtracted from each image. We determined the areas encompassed by gap junction staining (for each connexin) based on the number of pixels above threshold intensity using Image J [38] similarly to the approaches described previously [39, 40]. The extent of overlap of the two proteins in each double label pair was quantified using the Colocalization Threshold plug-in to the Image J software. A single Mander’s calculation produces two colocalization coefficients, M1 and M2, with values between 0 (no colocalization) and 1 (perfect colocalization).

### Extracellular space assessment

WGA is a lectin that binds to glycoconjugates and has been used to assess the abundance and distribution of cell membranes and the extracellular matrix in cardiac tissues [29, 36]. In normal and hypertrophic hearts, the distribution of WGA staining is similar to that of Masson’s trichrome, which is commonly used to detect collagen/fibrosis [29]. Tissue staining with fluorescent WGA was analyzed using Image J software (http://rsb.info.nih.gov/ij/). The area of fluorescence (WGA-reactive tissue) was determined after conversion to binary (black and white) images by applying the automatic threshold level of pixel intensity. Tissue area was calculated by subtracting areas not covered with tissue (“holes”) from the whole image area. The threshold for quantification of “hole area” was determined by side-by-side comparison of the binary and original color images, and the same threshold was used for all images. The percentage of WGA-reactive tissue was calculated by dividing fluorescent image area by total tissue area.

The same strategy was used to calculate the percentage of connexin or N-cadherin reactive tissue in atria or ventricle.

## Additional file

Additional file 1: Figure S1. Extracellular space is similar in micrographs of ventricular sections prepared from wild type C57BL/6J mice treated with RA or IH. Representative fluorescence photomicrographs are shown for sections of ventricle from RA (left) and IH (right) treated mice. Glycoconjugates within the extracellular spaces (and at plasma membranes) were localized by reaction of sections with WGA-Texas Red-X. Immunofluorescence images were analyzed using Image J as described in Material and Methods. The abundance and distribution of fluorescent staining appeared similar in both samples; moreover, it did not differ quantitatively (as presented in Results). Bar, 40 μm. (PDF 130 kb)

## Abbreviations

AF: Atrial fibrillation
Cx: Connexin
IH: Intermittent hypoxia
NOX2: NADPH Oxidase 2
OSA: Obstructive sleep apnea
qRT-PCR: Quantitative Reverse Transcription-Polymerase Chain Reaction
RA: Room air
ROS: Reactive oxygen species
SDS-PAGE: Sodium Dodecyl Sulfate Polyacrylamide Gel Electrophoresis
