# Supplementary material for: Intermittent hypoxia causes NOX2-dependent remodeling of atrial connexins
Source: BMC Cell Biol. 2017 Jan 17;18(Suppl 1):7. doi: 10.1186/s12860-016-0117-5 (PMC5267331; doi:10.1186/s12860-016-0117-5)
Supplement: Additional file 1: Figure S1. — Extracellular space is similar in micrographs of ventricular sections prepared from wild type C57BL/6J mice treated with RA or IH. Representative fluorescence photomicrographs are shown for sections of ventricle from RA (left) and IH (right) treated mice. Glycoconjugates within the extracellular spaces (and at plasma membranes) were localized by reaction of sections with WGA-Texas Red-X. Immunofluorescence images were analyzed using Image J as described in Material and Methods. The abundance and distribution of fluorescent staining appeared similar in both samples; moreover, it did not differ quantitatively (as presented in Results). Bar, 40 μm. (PDF 130 kb) [file 12860_2016_117_MOESM1_ESM.pdf]

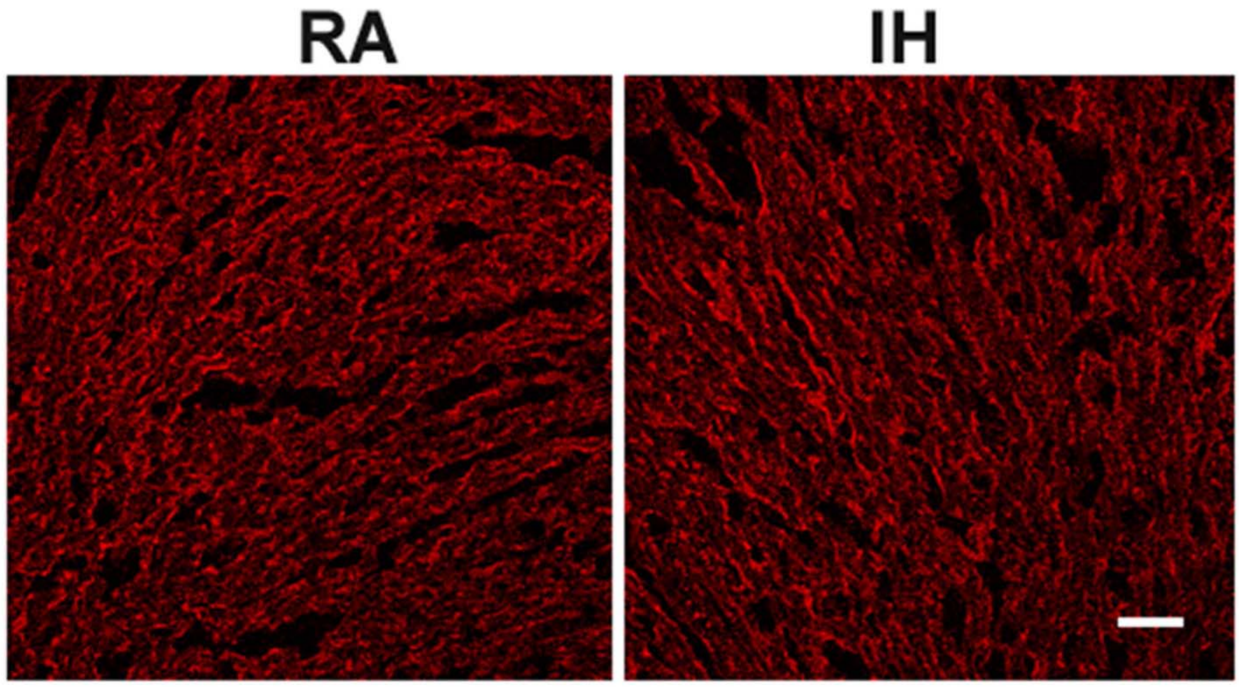

Figure S1. Extracellular space is similar in micrographs of ventricular sections prepared from wild type C57BL/6J mice treated with RA or IH. Representative fluorescence photomicrographs are shown for sections from RA (left) and IH (right) treated mouse ventricle. Glycoconjugates within the extracellular spaces (and at plasma membranes) were localized by reaction of sections with WGA-Texas Red-X. Immunofluorescence images were analyzed using Image J as described in Material and Methods. The abundance and distribution of fluorescent staining appeared similar in both samples; moreover, it did not differ quantitatively (as presented in Results). Bar, 40 µm.
